# Infrared Thermal Imaging and Blood and Faecal Cortisol Measurements for Stress Assessment in Rabbits During Blood Sampling

**DOI:** 10.3390/ani16172731

**Published:** 2026-09-02

**Authors:** Jana Doležalová, Gabriela Kadlecová, Vladimír Jekl, Monika Šebánková, Edita Jeklová, Eva Jánová, Petra Bakulová, Eva Voslářová, Kristýna Řeháková, Zuzana Tomiczková, Jan Chloupek

**Affiliations:** 1 Department of Physiology, Faculty of Veterinary Medicine, University of Veterinary Sciences Brno, 612 42 Brno, Czech Republic; dolezalovaja@vfu.cz (J.D.);; 2 Department of Animal Protection and Welfare & Veterinary Public Health, Faculty of Veterinary Hygiene and Ecology, University of Veterinary Sciences Brno, 612 42 Brno, Czech Republic; lukesovag@vfu.cz (G.K.); voslarovae@vfu.cz (E.V.); 3 Department of Pharmacology and Pharmacy, Faculty of Veterinary Medicine, University of Veterinary Sciences Brno, 612 42 Brno, Czech Republic; valdimirjekl@gmail.com (V.J.);; 4 Infectious Diseases and Preventive Medicine, Veterinary Research Institute v.v.i., Hudcova 296/70, 621 00 Brno, Czech Republic; 5 Department of Forest Ecology, Faculty of Forestry and Wood Technology, Mendel University in Brno, Zemedelska 1665/1, 613 00 Brno, Czech Republic; 6 Small Animal Clinical Laboratory, Faculty of Veterinary Medicine, University of Veterinary Sciences Brno, Palackého 1946/1, 612 42 Brno, Czech Republic

**Keywords:** stress, cortisol, body temperature, rabbit, thermal imaging, faeces

## Abstract

**Background:** This study evaluated physiological responses to repeated handling and blood collection in laboratory rabbits using infrared thermography, serum cortisol, and faecal cortisol metabolites (FCMs), and assessed responses in non-handled rabbits housed in the same room. **Methods**: Sixteen healthy adult rabbits were randomly allocated to experimental (n = 8) and non-handled control (n = 8) groups. Experimental rabbits underwent nine blood collections over 5 h as part of a pharmacokinetic study, with periocular surface temperature measured by infrared thermography before each collection. Controls underwent thermography at identical time points without handling or blood sampling. Serum cortisol was measured in experimental rabbits, whereas FCMs were analysed in both groups. **Results**: Following the first blood collection, periocular temperature increased by 1.0 °C in experimental and 1.3 °C in control rabbits. Experimental serum cortisol increased from 13.6 ± 5.5 to 23.7 ± 7.3 ng/mL at 30 min and declined to 8.1 ± 2.1 ng/mL at final sampling. FCMs increased approximately 9 h after the first collection in both groups and remained elevated for up to three days in experimental rabbits. **Conclusions**: Repeated handling and blood collection elicited measurable physiological responses. Changes in controls suggest that procedure-related environmental cues may also influence nearby non-handled rabbits.

## Simple Summary

Laboratory rabbits are widely used in biomedical research, and routine procedures such as handling and blood collection may induce physiological stress responses that affect animal welfare and experimental outcomes. This study investigated the effects of repeated blood collection on stress-related physiological responses in rabbits and whether non-handled rabbits housed in the same room also exhibited measurable changes. Sixteen rabbits were included. Eight animals underwent repeated blood collection over 5 h, while eight non-handled control rabbits remained in the same room. Physiological responses were assessed using infrared thermography to measure periocular surface temperature, serum cortisol in the experimental group, and faecal cortisol metabolites in both groups. Rabbits subjected to repeated blood collection showed rapid increases in periocular surface temperature and serum cortisol concentrations, while faecal cortisol metabolites increased later and remained elevated for up to three days. Control rabbits also showed transient physiological changes despite the absence of direct handling, suggesting that exposure to procedure-related environmental cues may influence nearby animals. These findings highlight the importance of minimising unnecessary disturbance during laboratory procedures and considering housing conditions during experimental procedures. Infrared thermography may also provide a useful non-invasive method for monitoring acute physiological responses when used alongside established stress indicators.

## 1. Introduction

Domestic rabbits are widely used as experimental animals because of their well-studied physiology [1,2] and the wide range of diseases that have been systematically described and further investigated in experimental settings [3]. Additionally, rabbits serve as a valuable model organism for studies of human diseases [4,5,6] and for the evaluation of pharmaceutical efficacy in both human and veterinary medicine [7,8,9].

During experimental procedures, rabbits may experience significant stress, either as a result of housing conditions or handling interventions. Previous studies have shown that these stressors can affect physiological and behavioural parameters, including heart rate, behaviour, and endocrine responses such as corticosterone and prolactin levels [10]. These changes may affect the validity and interpretability of experimental outcomes and should therefore be minimised [11]. Additionally, housing systems can significantly influence animal behaviour. The occurrence of stereotypic behaviours has been well documented and is closely associated with stress and reduced welfare in rabbits [12,13,14].

Several methods are available for assessing stress in rabbits, including measurement of stress-related hormones in saliva, hair, faeces, or blood [15,16,17,18,19,20]. However, saliva and blood sampling have significant limitations, as the procedures themselves may induce stress, thereby influencing the measured parameters [20]. Although early habituation to handling can reduce stress responses during sampling, painful or invasive procedures associated with certain experimental interventions may cause more persistent or long-term stress effects [21].

In accordance with the widely accepted principles of the 3R concept (Replacement, Reduction, Refinement), substantial efforts have been made to improve the housing and management of experimental animals and to minimise any negative impact on their welfare (Act No. 246/1992 Coll. of the Czech Republic). To enhance the welfare of animals in research facilities, non-invasive methods are recommended, as they reduce stress and data bias [22]. In this context, infrared thermal imaging is a promising alternative for collecting physiological data without direct physical interaction with the animals [20,23].

Stress-induced changes in body surface temperature are mediated by activation of the sympathetic nervous system and the hypothalamic–pituitary–adrenal (HPA) axis. Acute stress rapidly alters peripheral blood perfusion through sympathetic vasoconstriction or vasodilation, leading to measurable changes in skin temperature before endocrine responses such as glucocorticoid secretion reach their peak. These thermoregulatory responses have been described in several mammalian species and provide a physiological basis for using infrared thermography as a non-invasive indicator of acute emotional and physiological stress. Changes are particularly evident in highly vascularised, minimally haired body regions, including the periocular area, nasal region and ear pinnae, where temperature closely reflects alterations in local blood flow rather than core body temperature [24,25,26,27].

Although infrared thermography has been increasingly used for welfare assessment in domestic and laboratory animals, evidence supporting its use to detect acute stress responses in rabbits remains limited. Previous rabbit studies have primarily focused on clinical applications, thermoregulation, pain assessment or environmental influences, while only a few have investigated acute psychological or handling-related stress under controlled experimental conditions. Furthermore, little information is available regarding the relationship between thermal responses and established endocrine biomarkers, particularly faecal cortisol metabolites (FCMs), which provide an integrated measure of hypothalamic–pituitary–adrenal axis activation without the confounding effects of blood sampling. Consequently, the diagnostic value of infrared thermography as a refinement tool for non-invasive stress assessment in laboratory rabbits has not yet been fully established [24,25,26,27].

The aim of this study was to determine whether infrared thermography can detect acute stress responses in laboratory rabbits exposed to two commonly encountered experimental stressors that differ in intensity and nature (blood sampling versus non-handled same-room controls). We hypothesised that acute stress would induce rapid changes in periocular surface temperature mediated by autonomic vascular responses. By comparing infrared thermography with conventional hormonal biomarkers, this study aims to evaluate whether thermal imaging can serve as a promising non-invasive method refinement tool for the non-invasive assessment of rabbit welfare during experimental procedures.

## 2. Materials and Methods

### 2.1. Animals

Sixteen New Zealand White Rabbits, strain 052, age 8 months, weight (median 3.24, range min-max 2.94–3.67 kg), sex ratio 1:1, were housed individually in two-tier wire-mesh cages located within the same experimental room. Experimental rabbits were purchased from an accredited laboratory animal supplier (Anlab, Prague, Czech Republic). Each cage contained a solid/wire mesh floor and a solid elevated platform, and opaque partitions separated adjacent cages, preventing direct visual contact between animals. Nevertheless, auditory and olfactory cues generated during routine handling and blood collection remained accessible to all animals housed in the room. Direct physical contact between rabbits was prevented throughout the study.

The rooms were maintained at a temperature of 18–20 °C with 45–55% relative humidity, ventilation of approximately 15 air changes per hour, and a 12 h light/dark cycle. These parameters remained stable throughout the entire experimental study.

The rabbits had free access to water and were fed ad libitum with a complete pelleted diet (Biostan KBO, Biokron, Czech Republic). The chemical composition of BIOSTAN KBO on an as-fed basis was 88.9% dry matter, 14.8% crude protein, 3.7% crude fat, 13.9% crude fibre, 30.7% neutral detergent fibre (NDF), 16.7% acid detergent fibre (ADF), approximately 4.2% acid detergent lignin (ADL), 22.7% starch, 3.3% total sugars, and 9.5% crude ash. These values are consistent with a moderate-energy, high-fibre diet formulated for breeding rabbits outside the lactation period. The calculated mineral composition included approximately 1.41% calcium, 0.71% phosphorus, 1.14% potassium, 0.21% magnesium, and at least 0.14% sodium, resulting in an estimated calcium-to-phosphorus ratio of approximately 2.0:1. Based on the ingredient composition, the estimated digestible energy concentration for rabbits was 9.8–10.6 MJ/kg (2340–2530 kcal/kg), with an estimated metabolisable energy content of 8.7–9.5 MJ/kg (2080–2270 kcal/kg). These values correspond well to recommendations for maintenance and reproductive rabbits and reflect the relatively high proportion of structural carbohydrates derived from dehydrated alfalfa, wheat bran, sunflower meal, and malt sprouts. The feed formulation also included a commercial amino acid, vitamin, and trace mineral premix (1.0% of the diet) together with the probiotic preparation PROBIOSTAN E10 (0.2%). Before enrolment, all rabbits underwent a complete physical examination performed by an experienced veterinarian/specialist to confirm the absence of clinical disease or other conditions that might influence physiological stress responses. Haematology, plasma chemistry, urinalysis and faecal flotation parasitology were performed to exclude potential subclinical disease. Only clinically healthy animals were included in the study. After the acclimation period of three weeks, rabbits were randomly allocated into two groups (experimental group, n = 8, sex ratio 1:1; and control group, n = 8, sex ratio 1:1). During the three-week acclimation period, all rabbits underwent a daily habituation procedure lasting approximately 15 min. Habituation was performed by two same experienced persons throughout the study using calm, low-stress handling techniques. Animals were approached quietly without sudden movements or loud noises and were allowed to orient towards the handler before being gently removed from the cage. Gentle tactile contact preceded lifting, and restraint was kept to the minimum necessary. Rabbits were returned immediately to their home cage after each habituation session. No painful procedures were performed during the habituation period. The aim was to familiarise the animals with human presence, handling and restraint before the experimental procedures.

The study formed part of a previously approved pharmacological experiment, and the number of animals was determined in accordance with the Reduction principle of the 3Rs. A formal a priori power analysis was not performed because sufficiently reliable preliminary estimates of the expected size effect for infrared thermographic and FCM responses in rabbits were unavailable. Eight animals were therefore included in each group. Standardised between-group effect sizes were subsequently calculated as Cohen’s d using pooled standard deviations, together with 95% confidence intervals. Achieved statistical power was estimated for two-sided comparisons at α = 0.05. The calculated power varied considerably among time points and was generally low for small-to-moderate between-group effects.

All procedures and animal experiments were performed in full compliance with the ARRIVE guidelines and the European Community Council Directive (2010/63/EU). The study was approved by the Institutional Ethics Committee of the University of Veterinary Sciences Brno (approval project number 13–2021).

### 2.2. Study Protocol

**Figure 1:**
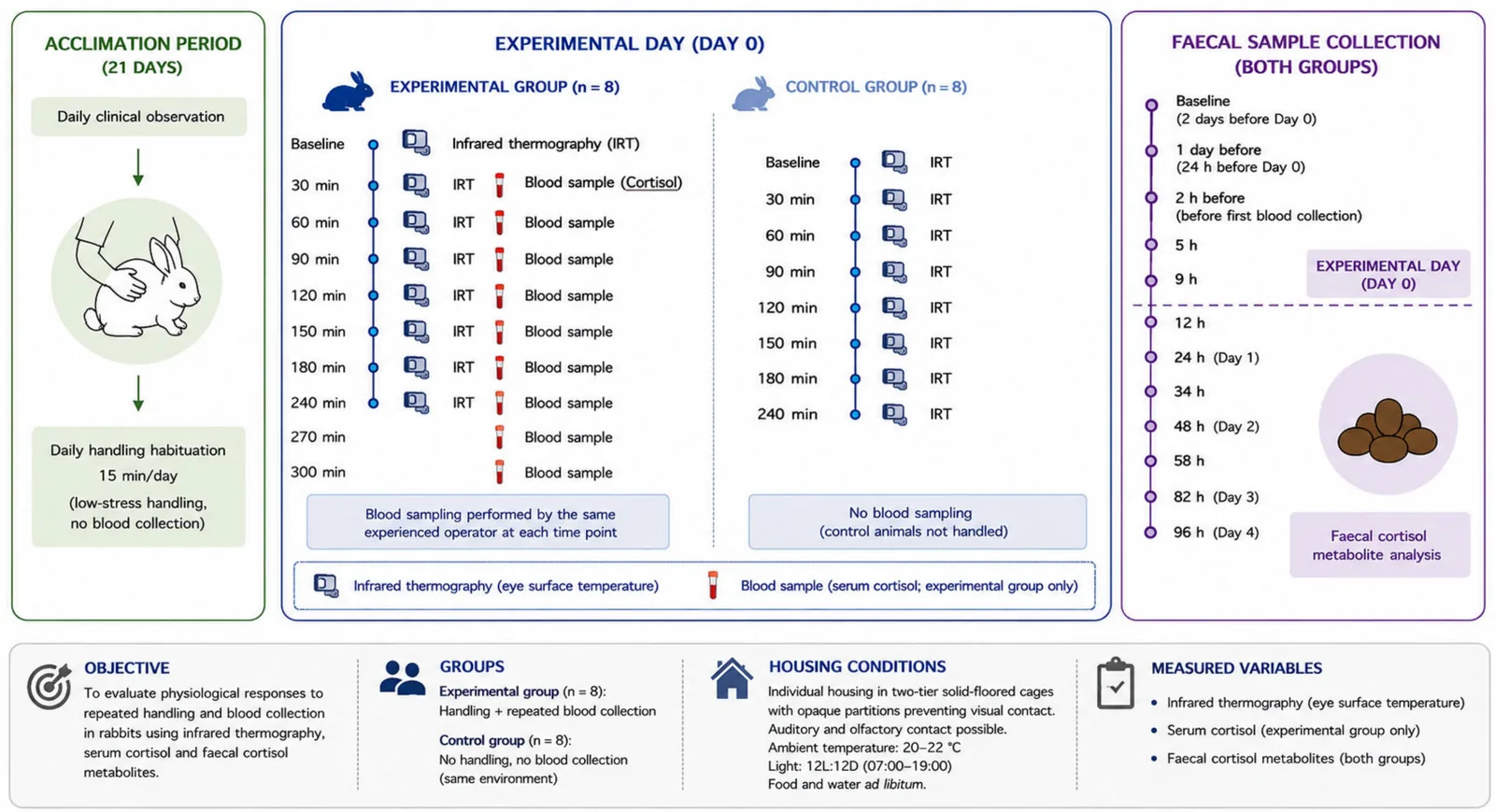
Experimental design and sampling timeline (AI-generated image, only data from the present study was used).

The study consisted of three consecutive phases: (i) a three-week acclimation period including daily habituation to routine handling, (ii) the experimental day involving repeated blood collection and infrared thermographic measurements, and (iii) post-experimental monitoring of *FCM* concentrations for 96 h. On the experimental day, thermal images were obtained immediately before each scheduled blood collection in the experimental group and at identical time points in the control group. Blood samples were collected only from rabbits assigned to the experimental group, whereas faecal samples were collected from both groups according to the predefined sampling schedule. Experimental design and sampling timeline are summarised in Figure 1.

Rabbits underwent a 21-day acclimation period including daily handling habituation before the experimental procedures. Infrared thermographic images were acquired immediately before each scheduled blood collection in the experimental group and at identical time points in the control group. Blood samples for serum cortisol analysis were collected only from the experimental group to avoid introducing handling-related stress in non-handled same-room control (control group) animals. Faecal samples were collected from both groups before and after the experiment to assess delayed changes in *FCMs*.

Infrared thermographic imaging of the rabbit head was performed using a Testo 890-2 thermal camera (Testo SE & Co. KGaA, Germany, with a resolution of 640 × 480 pixels, a thermal sensitivity of less than 0.04 °C, and a measurement range of −30 to 100 °C. Images were captured at a 90° angle from a distance of 0.3 m. Temperature measurements used in this study were taken from the medial canthus of the eye to minimise the influence of fur on thermal readings. Data were extracted from infrared thermographic images using IRSoft software (Testo SE & Co. KGaA, Germany). Baseline periocular surface temperature was recorded 24 h before blood collection, and these measurements were used as reference values for subsequent analyses. Then, the periocular surface body temperature was measured immediately after the first blood collection and then at 30, 60, 180, and 240 min of the experiment.

Blood samples (3 mLs) were collected from the central auricular artery with a 25-gauge needle and a blood container. Each rabbit was restrained by an experienced handler within a towel, with its ears exposed. The chlorhexidine solution (SkinMed spray, Cymedica, Czech Republic) was used for disinfection, and blood samples were obtained after the solution had evaporated/dried to avoid contamination of the final samples. The first blood collection corresponds to the 0 min time point in the experimental protocol, and serum cortisol levels from these samples serve as the baseline. The following blood samples were obtained at 30, 60, 120, 180, 210, 240, 270, and 300 min after the first blood sampling. Blood was obtained at these time points for the pharmacological study. After coagulation and centrifugation (3000 RCF, 5 min), the serum was stored at −20 °C until the following cortisol measurements.

Fresh faecal samples (approximately 2 g per sample) were collected from all rabbits once daily, two days before blood collection, as well as 2 h before sampling. The faecal samples were collected at the next 9 time points (5, 9, 12, 24, 34, 48, 58, 82, and 96 h from the start of the experiment/blood collection). All samples were placed in sealed plastic bags and stored at −20 °C until analysis.

Animals in the control group were housed in the same room as the experimental group during blood collection, and temperature was measured using a thermometer at the same time points. These animals remained in their cages and were not subjected to handling or experimental manipulation.

### 2.3. Blood and Faecal Cortisol Metabolite Measurement

Cortisol concentration in faecal and serum samples was measured using the DetectX^®^ Cortisol Enzyme Immunoassay Kit (Arbor Assays, Michigan, IN, USA) according to the manufacturer’s instructions. Absorbance was measured at 450 nm using a spectrophotometer reader (BioTek Elx808, Biotek©, Shoreline, WA, USA). The analysis was repeated if the sample’s intra-assay coefficient of variability (CV) exceeded 10% and the inter-assay CV 15%. The cross-reactivity of the cortisol antibodies used was 100% for cortisol, 18.8% for dexamethasone, 7.8% for prednisolone (1-dehydrocortisol), 1.2% for corticosterone and cortisone, and less than 0.1% for progesterone, oestradiol, cortisol 21-glucuronide, 1α-hydroxycorticosterone, and testosterone.

Blood samples were diluted two times with assay buffer prior to analysis, and cortisol concentrations were calculated in ng/mL in serum.

Frozen faecal samples were dried in an air dryer (Memmert, Bremen, DE, USA) at 50 °C for 2–6 h. The dried faecal material was then ground into a fine powder, and aliquots (up to 0.11 g) were weighed into 15 mL vial tubes. For the extraction of steroid hormones, 80% ethanol was added (1 mL of ethanol per 0.1 g of dried faeces) to each sample. Samples were vortexed for 30 min, and then centrifuged at 5000 rpm for 15 min at 4 °C. The supernatant was collected and stored at −20 °C until immunoassay analysis. All samples were diluted 10 times before the analysis. The final concentration of FCMs was calculated in ng/g of dried faeces. Before analysis, all samples were diluted 1:10. Final FCM concentrations were expressed as ng/g of dried faeces.

### 2.4. Statistical Analysis

Periocular surface temperature, blood cortisol, and FCM concentrations were expressed as mean ± standard deviation (SD) for all measurements. All statistical analyses were performed using STATISTICA, version 14.3 (TIBCO Software Inc., Palo Alto, CA, USA). A *p*-value < 0.05 was considered statistically significant. Before statistical testing, continuous variables were visually inspected using histograms and quantile–quantile plots. The normality of data distribution was evaluated using the Shapiro–Wilk test, and homogeneity of variances was assessed using Levene’s test. Variables that did not meet the assumptions of normality were analysed using non-parametric methods.

Over time, periocular surface temperature was measured repeatedly in the same animals. To evaluate the effects of experimental group (experimental vs. control), sampling time, and their interaction on periocular surface temperature, a General Linear Model (GLM) for repeated measurements was applied. Rabbit identity was considered the repeated observational unit, thereby accounting for the non-independence of repeated measurements obtained from the same animal.

Changes in serum cortisol concentrations during the experiment were evaluated only within the experimental group because repeated blood sampling was not performed in control rabbits. Serum cortisol concentrations at each sampling time were compared with baseline values (time 0) using the paired Wilcoxon signed-rank test.

Differences in FCM concentrations between the experimental and control groups at each sampling time point were assessed using the non-parametric Mann–Whitney U test. In addition, to evaluate individual stress responses, within-subject changes in FCMs before and after the blood collection procedure were analysed using the paired Wilcoxon signed-rank test.

## 3. Results

### 3.1. Evaluation of Stress Response Using Temperature Data

**Figure 2:**
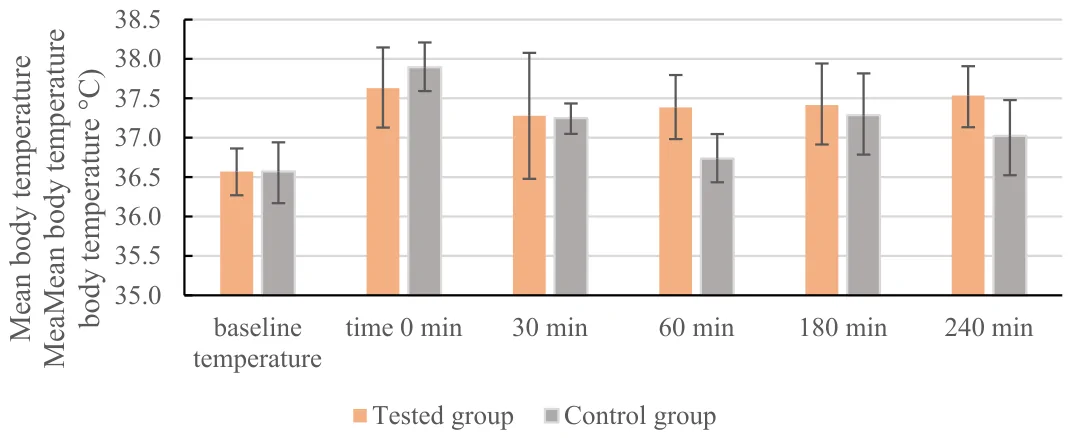
Mean periocular surface temperature (±SD) measured at the medial canthus of the eye in rabbits before (baseline) and during blood collection (immediately after sampling at time 0, and at 30, 60, 180, and 240 min after the first blood collection). The tested group undergoes blood sampling, while the control group remains in the same room and is measured at the same time points without handling.

Baseline mean values of periocular surface temperature were 36.6 ± 0.3 °C in the experimental group and 36.6 ± 0.4 °C in the non-handled same-room control group. After the first blood collection, body temperatures significantly increased by 1.0 °C in the experimental group and 1.3 °C in the control group compared with baseline values (Wilcoxon signed-rank test, z = 2.060; z = 2.383, respectively; see Figure 2). The between-group difference in change from baseline was −0.26 °C (95% CI, −0.86 to 0.33 °C). Except for first sampling, animals in the experimental group tended to have higher periocular surface temperatures and higher values on all other measurements than the control group; however, these differences were not statistically significant (GLM, χ^2^ = 2.798; *p* = 0.09). In both groups, surface temperature was significantly affected by the sequence of blood collection (GLM, X^2^ = 20.194; *p* = 0.0005).

### 3.2. Blood Cortisol Values

**Figure 3:**
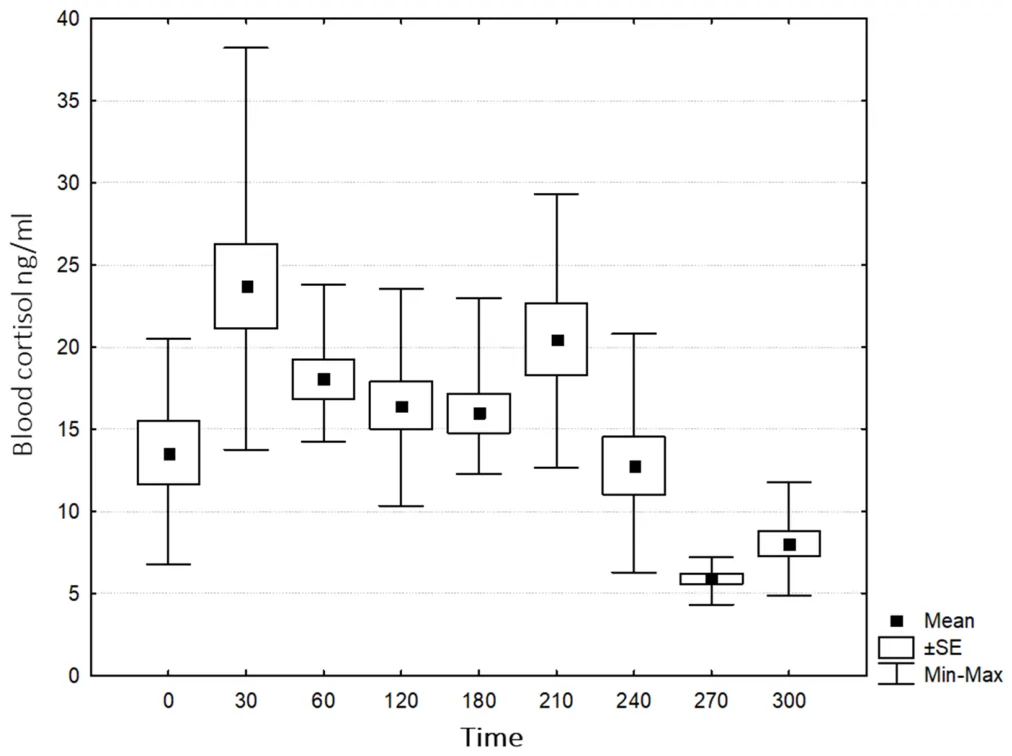
The distribution and mean values of blood cortisol in the experimental group during the blood collection and handling. Values are presented as mean ± standard error (SE), with minimum (Min) and maximum (Max) values indicated.

Intensive handling and repeated blood collection were associated with an initial increase in serum cortisol concentrations in both groups, with significantly elevated levels at 30 min after the first sampling (*p* < 0.05). Serum cortisol levels then decreased, returning to baseline by 240 min. At later time points, cortisol levels at 270 and 300 min were significantly lower than at baseline (time 0) (Wilcoxon signed-rank test: 270 min, z = 2.521, *p* = 0.012; 300 min, z = 2.100, *p* = 0.036; see Table 1 and Figure 3).

### 3.3. Faecal Cortisol Metabolites

Compared with baseline values prior to blood collection, FCM levels in the tested group increased significantly over the 9 h period following the start of blood collection, reaching 2.4 times baseline levels (Wilcoxon test, z= 2.521, *p* = 0.012). A significant increase was also observed in the control group, where faecal cortisol metabolite concentrations increased 1.9 times in comparison with baseline levels (Wilcoxon signed-rank test: z = 2.197, *p* = 0.028). Compared to baseline values, elevated cortisol metabolite levels persisted for up to three days (96 h) following the intervention and intensive handling. On the second and third days, concentrations tended to be lower in the morning and higher in the afternoon (24 h vs. 34 h; 48 h vs. 58 h; see Table 2).

## 4. Discussion

Physiological and pathological conditions, including acute stress, activate the sympathetic nervous system and the hypothalamic–pituitary–adrenal (HPA) axis, resulting in the release of catecholamines and glucocorticoids. Sympathetic activation induces rapid changes in peripheral vascular tone, leading to redistribution of cutaneous blood flow and measurable alterations in body surface temperature. Consequently, infrared thermography has become an increasingly recognised non-invasive method for assessing acute physiological responses to stress in a variety of animal species [24,25]. However, thermal responses reflect autonomic regulation rather than direct endocrine activity and are influenced by several biological and environmental factors, including the anatomical region examined, ambient conditions and species-specific thermoregulatory mechanisms [23,24,25].

In the present study, repeated handling and blood collection were associated with rapid increases in periocular surface temperature measured at the medial canthus of the eye. Rabbits subjected to repeated blood sampling showed an increase of approximately 1.0 °C above baseline, whereas rabbits housed in the same room but not directly handled showed an initial increase of approximately 1.3 °C. Although the initial increase was numerically greater in the control group, subsequent measurements tended to remain higher in the experimental group. However, these between-group differences did not reach statistical significance and should therefore be interpreted cautiously. The similar immediate responses observed in both groups suggest that factors associated with the experimental procedures, rather than direct handling alone, may have contributed to the physiological changes observed in nearby animals.

Blood cortisol concentrations, measured only in the experimental group, also demonstrated activation of the physiological stress response. Mean serum cortisol concentrations increased from 13.6 ± 5.5 ng/mL at baseline to 23.7 ± 7.3 ng/mL 30 min after the first blood collection and subsequently declined, returning to values comparable with baseline by 240 min. Interestingly, cortisol concentrations at 270 and 300 min were significantly lower than baseline values. This decline may reflect recovery following the initial stress response, negative feedback regulation of the HPA axis, circadian variation, or partial habituation to repeated restraint and sampling. Because no autonomic or behavioural indicators of stress were collected, the relative contribution of these mechanisms cannot be determined. Consequently, the present findings demonstrate measurable physiological responses associated with repeated handling and blood collection but do not allow definitive conclusions regarding the persistence of stress or the development of habituation. In the present study, periocular surface temperature increased by more than 1 °C above baseline immediately after the first blood collection in the experimental group and by approximately 1.3 °C in the control group, exceeding the approximately 0.4 °C increase reported in rabbits exposed to stress during natural mating [26]. Although the initial increase was numerically greater in the control group, subsequent temperature values tended to remain higher in the experimental group. Because these differences were not statistically significant, they should be interpreted with caution. The similar immediate responses observed in both groups suggest that factors associated with the experimental procedures, rather than direct handling alone, may have contributed to the physiological responses observed in nearby animals. Thermal responses to acute stress have also been reported in other species, including horses (3.5 °C) [27], dogs (0.25 °C) [28], goats (1.2 °C) [29], blue tits (4.6 °C) [30], and chickens (1.1 °C) [31]. However, direct comparison between studies should be made cautiously because thermal responses depend on species-specific thermoregulatory mechanisms, the anatomical region examined, the nature of the stressor, and differences in infrared thermography protocols. In contrast to rabbits, some species, including reindeer [20] and cattle [32], exhibit an initial decrease in eye temperature following acute stress, probably reflecting species differences in autonomic regulation of peripheral blood flow resulting in changes in cutaneous perfusion [28].

During the subsequent measurements (30–240 min), periocular surface temperature in the experimental group decreased after the initial peak and then remained relatively stable, without significant differences compared with values recorded immediately after the first blood collection (see Figure 2). Although this pattern may indicate sustained physiological activation during repeated restraint and blood sampling, the absence of significant differences between consecutive measurements does not allow conclusions regarding a continuous stress response. In the control group, periocular surface temperature declined by 1.15 °C during the first hour, followed by moderate fluctuations throughout the observation period. Because environmental conditions and potential sensory stimuli were not quantified, the mechanisms underlying these fluctuations cannot be determined. Nevertheless, rabbits housed in the same room remained exposed to procedure-related auditory and olfactory cues associated with repeated handling and blood collection of neighbouring animals, which may have contributed to the observed physiological responses despite the absence of direct handling.

The first measurement likely represented the animals’ response to a novel experimental situation. During subsequent observations, periocular surface temperature declined but generally remained above baseline until the end of the monitoring period. Although repeated exposure to routine handling has been reported to attenuate physiological stress responses in dogs [28] and rabbits [33], the present study cannot determine whether a similar habituation process occurred because behavioural indicators and autonomic measures were not assessed. Furthermore, the repeated restraint and multiple blood collections performed in the experimental group represent substantially different stimuli from routine handling alone. A transient decrease in periocular surface temperature observed 30 min after the initial blood collection in both groups is consistent with previous observations in rats [34] and blue tits [30]. Nevertheless, periocular surface temperature returned to baseline values in all rabbits during the monitoring period, indicating that the physiological response associated with the experimental procedures was transient. Similar transient behavioural responses have previously been reported in rabbits following tactile interaction with unfamiliar humans, including increased salivary corticosterone concentrations, tense body posture and ears held backwards, with behavioural recovery occurring within 4–8 min [18]. These observations support the hypothesis that rabbits exhibit measurable but reversible physiological and behavioural responses to acute experimental procedures.

Serum cortisol concentrations, measured only in the experimental group, confirmed activation of the physiological stress response, with peak concentrations occurring within 30 min after the first blood collection. Interestingly, cortisol concentrations declined below baseline values at 270 and 300 min. This finding may reflect recovery following the initial stress response, negative feedback regulation of the hypothalamic–pituitary–adrenal axis, circadian variation, or partial adaptation to repeated restraint and sampling; however, the relative contribution of these mechanisms cannot be determined from the present data. Elevated FCM concentrations remained detectable for up to three days in the experimental group. This prolonged pattern cannot be explained solely by the intestinal passage of metabolites resulting from a single brief cortisol peak. A more likely explanation is that repeated restraint and nine blood collections performed over five hours induced cumulative or recurrent activation of the HPA axis, with additional glucocorticoid secretion continuing during the recovery period. In addition, FCMs represent an integrated measure of glucocorticoid secretion over several hours rather than an instantaneous endocrine response, reflecting both endocrine activity and gastrointestinal transit time rather than circulating cortisol concentrations at the time of sampling [35,36,37,38,39,40]. In rabbits, interpretation of FCMs is further complicated by their unique digestive physiology. The proximal colon separates digesta into hard faeces and soft caecotrophs, which are normally reingested directly from the anus (caecotrophy) [41,42]. After reingestion, caecotrophs, protected by a mucus coating formed in the proximal colon, pass through the stomach with limited exposure (3–6 h) to gastric digestion before entering the small intestine allowing reutilisation of microbial protein, vitamins and other nutrients [41,42]. Although there is currently no direct evidence that glucocorticoid metabolites are reabsorbed or subsequently re-excreted following caecotrophy, this physiological recycling process may influence gastrointestinal transit time and contribute to the temporal dispersion and interindividual variability of FCM excretion. Consequently, the prolonged elevation observed in the present study probably reflects a combination of sustained HPA-axis activation, prolonged gastrointestinal passage, and species-specific digestive physiology. Further pharmacokinetic validation studies are needed to determine the contribution of caecotrophy to FCM kinetics in rabbits [39,40]. Rabbits in the present study underwent daily handling during the acclimatisation period; repeated exposure alone does not necessarily result in effective habituation. Current evidence indicates that successful habituation depends on the quality of human–animal interactions rather than their frequency [22]. Low-stress habituation protocols based on gradual exposure, predictable handling, voluntary approach, and positive reinforcement have been shown to reduce fear responses and physiological stress in rabbits and several domestic species. In contrast, repeated restraint without positive reinforcement may maintain or even enhance HPA-axis activation in susceptible individuals. Future studies should therefore evaluate whether structured low-stress habituation protocols incorporating positive reinforcement-based habituation before experimental procedures further reduce physiological stress responses measured by infrared thermography and FCMs. Our findings appear to differ from those reported by Urbanová et al. [33], who observed reduced stress responses in rabbits repeatedly handled during teaching sessions. However, the two studies addressed different types of handling. In the study by Urbanová et al. [33], habituation involved repeated human contact and routine handling without repeated invasive procedures, whereas rabbits in the present study underwent repeated restraint associated with nine blood collections over a five-hour period. Consequently, the physiological responses observed in our study likely reflect the combined effects of handling, restraint and repeated venipuncture rather than handling alone. Moreover, habituation is stimulus-specific and depends on the biological significance, predictability and intensity of the stimulus. Animals may successfully habituate to gentle handling while continuing to exhibit physiological stress responses to repeated invasive procedures. Similar observations have been reported in laboratory and domestic animals, where habituation reduces responses to routine husbandry but has a limited effect on repeated noxious or aversive interventions [22,43,44,45]. Therefore, the persistent endocrine response observed in the present study does not necessarily indicate unsuccessful habituation but rather reflects the cumulative physiological burden associated with repeated blood sampling.

Some limitations should be considered when interpreting the present findings. First, the study included a relatively small number of rabbits of a single breed and age category, which may limit the extrapolation of the findings to other rabbit populations. Second, behavioural and complementary physiological indicators (heart and respiratory rate) of stress were not evaluated concurrently with physiological measurements, precluding direct comparison between behavioural and endocrine responses. Third, gastrointestinal transit time and the kinetics of FCM excretion were not directly assessed; therefore, the mechanisms underlying the prolonged elevation of FCMs remain speculative. In addition, although rabbits underwent a habituation period before the experiment, the effectiveness of this protocol was not formally evaluated using behavioural or physiological endpoints. Periocular surface temperature was measured only at predefined time points and did not account for potential diurnal variation. Finally, core body temperature was not measured. Although rectal temperature may provide a more direct estimate of core temperature, this procedure was intentionally avoided because the additional handling required would have introduced a substantial source of stress, particularly in the control animals, thereby compromising the primary objective of the study. Also, blood sampling itself, is a stressor, which influences the HPA axis. Furthermore, unlike some avian species in which acute stress induces an initial decrease in superficial temperature due to peripheral vasoconstriction [38], this response has not been consistently demonstrated in rabbits. No correction for multiple comparisons was applied; therefore, the number of within- and between-group comparisons may have increased the risk of type I error. This should be considered when interpreting individual statistically significant findings, particularly those close to the significance threshold.

## 5. Conclusions

In conclusion, repeated handling and blood sampling induced measurable physiological stress responses in laboratory rabbits, demonstrated by increases in periocular surface temperature, serum cortisol, and FCM concentrations. From a welfare perspective, the present findings support the use of infrared thermography as a refinement tool for laboratory rabbits. Because thermal imaging enables repeated physiological assessment without physical contact, it has the potential to reduce handling-associated stress and improve data quality in longitudinal studies. Physiological responses observed in rabbits housed in the same room showed that experimental procedures also influence nearby animals, emphasising the importance of minimising environmental disturbance during laboratory experiments. If experimental procedures must be performed in the same room where control animals are housed, the potential impact on physiological measurements should be carefully considered, as animals not subjected to direct handling may nevertheless develop measurable stress responses following exposure to procedure-related auditory, visual, and olfactory stimuli.

## Figures and Tables

**Table 1:** Mean values (±SD) of serum cortisol levels in the experimental group. Differences between each sampling time point and baseline (time 0) are assessed using the Wilcoxon signed-rank test (z and *p* values). Statistically significant differences are marked by an asterisk. Mean difference was calculated between cortisol at time 0 and the appropriate time point; the lower and upper CIs indicated the 95% confidence interval for the difference between means.

Time/min.	0	30	60	120	180	210	240	270	300
Serum cortisol levels (ng/mL, mean ± SD)	13.6 ± 5.5	23.7 ± 7.3	18.1 ± 3.4	16.4 ± 4.1	16.0 ± 3.4	20.5 ± 6.2	12.8 ± 4.9	5.9 ± 0.9	8.1 ± 2.1
**z**		2.521 *	1.820	1.540	1.260	1.960 *	0.560	2.521 *	2.100 *
***p***		0.012 *	0.069	0.123	0.208	0.050 *	0.575	0.012 *	0.036 *
**Mean difference**		10.117	4.477	2.868	2.388	6.913	−0.799	−7.686	−5.525
**CI lower**		4.604	−0.575	−1.459	−1.980	0.748	−7.431	−12.357	−10.064
**CI upper**		15.630	9.528	7.195	6.756	13.077	5.834	−3.015	−0.986

**Table 2:** Mean FCMs concentrations (ng/g of dried faeces) in the experimental and control groups before, during, and after blood sampling. Between-group differences at each time point were assessed using the Mann–Whitney U test (z and *p* values). Within-group changes relative to baseline (defined as the mean of the first two samples collected two days prior to the experiment) were analysed using the Wilcoxon signed-rank test (z and *p* values). Statistically significant differences are indicated by asterisks. Mean difference A was calculated between the tested and control groups at each time point; Mean difference B was calculated between cortisol at time 0 and the appropriate time point; CI lower and CI upper indicate the 95% confidence interval for the difference between means.

	Before Blood Collection			During and After Blood Collection (from Time 0 Minutes)								
Time of faecal collection	2 d	1 d	2 h	5 h	9 h	12 h	24 h	34 h	48 h	58 h	82 h	96 h
Tested group, mean ± SD	224.9 ± 42.0	285.7 ± 124.9	223.9 ± 71.5	356.3 ± 212.6	613.3 ± 138.5	460.9 ± 209.5	321.9 ± 107.7	428.3 ± 103.3	345.6 ± 11.7	445.2 ± 113.5	417.2 ± 126.6	288.6 ± 106.9
Control group, mean ± SD	250.1 ± 65.7	351.1 ± 92.5	161.6 ± 44.5	286.8 ± 148.2	562.5± 164.5	398.2 ± 137.4	265.6 ± 150.4	341.2 ± 59.7	303.4 ± 135.2	393.1 ± 106.9	391.3 ± 96.1	437.9 ± 141.2
Non−paired Mann–Whitney test—tested vs. control group												
Z	0.231	−1.041	1.967 *	0.579	−0.116	0.579	1.389	1.504	1.157	0.579	0.579	−1.967 *
*p*	0.817	0.298	0.049 *	0.563	0.908	0.563	0.165	0.132	0.247	0.563	0.563	0.049 *
Mean difference A	−25.212	−65.299	62.297	69.429	50.819	62.722	56.293	87.112	42.117	52.111	25.836	−149.346
CI lower	−85.862	−189.561	−5.407	−138.136	−118.057	−138.377	−88.162	−9.019	−95.481	−71.449	−101.069	−287.889
CI upper	35.437	58.963	130.001	276.994	219.695	263.821	200.749	183.242	179.715	175.671	152.741	−10.804
Wilcoxon signed−rank test—tested group												
Z	−	−	0.42	0.84	2.521 *	2.1 *	1.68	2.38 *	1.82	2.24 *	2.1 *	0
*p*	−	−	0.674	0.401	0.012 *	0.036 *	0.093	0.017 *	0.069	0.025 *	0.036 *	1
Mean difference B			−31.425	100.979	358.005	205.599	66.582	173.000	90.262	189.943	161.873	33.278
CI lower			−130.828	−98.986	249.240	−3.518	−18.458	43.479	−16.635	72.689	31.332	−106.743
CI upper			67.978	300.944	466.770	414.715	151.621	302.521	197.160	307.197	292.413	173.299
Wilcoxon signed−rank test—control group												
Z	−	−	2.366 *	0.845	2.197 *	1.352	1.183	1.014	0.338	1.69	1.521	1.859
*p*	−	−	**0.018**	0.398	0.028 *	0.176	0.237	0.31	0.735	0.091	0.128	0.063
Mean difference			−138.977	−13.706	261.930	97.621	−34.967	40.633	2.890	92.576	90.781	137.369
CI lower			−202.129	−154.263	93.365	−48.315	−183.484	−48.952	−120.115	−11.817	−12.914	−15.385
CI upper			−75.826	126.851	430.495	243.558	113.550	130.217	125.895	196.969	194.476	290.122

## Data Availability

The original contributions presented in this study are included in the article. Further inquiries can be directed to the corresponding author.
